# Differences in uterine and serum metabolome associated with clinical cure failure of metritis in dairy cows^†^

**DOI:** 10.1093/biolre/ioaf038

**Published:** 2025-02-23

**Authors:** Frederico Narciso de Souza Pereira, Aline Martelo Pereira, Klibs Neblan Galvão, Rafael Sisconeto Bisinotto, Caio Cesar Figueiredo

**Affiliations:** Department of Veterinary Clinical Sciences, Washington State University, Pullman, WA, USA; Department of Veterinary Clinical Sciences, Washington State University, Pullman, WA, USA; Department of Large Animal Clinical Sciences, D. H. Barron Reproductive and Perinatal Biology Research Program, University of Florida, Gainesville, FL, USA; Department of Large Animal Clinical Sciences, D. H. Barron Reproductive and Perinatal Biology Research Program, University of Florida, Gainesville, FL, USA; Department of Veterinary Clinical Sciences, Washington State University, Pullman, WA, USA

**Keywords:** uterine disease, metabolites, inflammation

## Abstract

This study investigated differences in uterine and serum metabolome associated with clinical cure failure of metritis in dairy cows. Metritis was diagnosed in lactating Holstein cows from two Florida dairies and defined by the presence of fetid, watery, reddish-brown vaginal discharge from 4 to 12 days postpartum (dpp). Cows with metritis (*n* = 24) were paired with cows without metritis of similar parity and dpp (*n* = 24). On the day of metritis diagnosis (day 0), all cows with metritis received antimicrobial therapy. The continued presence of the fetid, watery, reddish-brown discharge on day 5 (*n* = 16) was defined as clinical cure failure, whereas clinical cure was defined by its absence (*n* = 8). Metabolome analyses of uterine lavage (days 0 and 5) and serum samples (day 0) were conducted using untargeted gas chromatography time-of-flight mass spectrometry. Normalized data were analyzed using partial least squares–discriminant analysis and ANOVA, adjusting *P*-values for multiple comparisons. Differences in the uterine metabolome on day 0 associated with clinical cure failure were linked to carbohydrate, amino acid, and lipid metabolism. Greater concentrations of arachidonic acid, ribose, and glutaric acid were associated with clinical cure failure, suggesting a greater degree of tissue lesion and inflammation. No differences in the serum metabolome were associated with cure failure. No differences in uterine metabolome were associated with clinical cure failure on day 5. The findings suggest that clinical cure failure is associated with a greater uterine inflammatory process that did not persist until cure assessment day.

## Teaser

Clinical cure failure in dairy cows with metritis is linked to distinct uterine metabolome profiles, inflammatory processes, and energy metabolism present on the day of metritis diagnosis but not on cure assessment day.

## Graphical Abstract

**Graphical Abstract:**
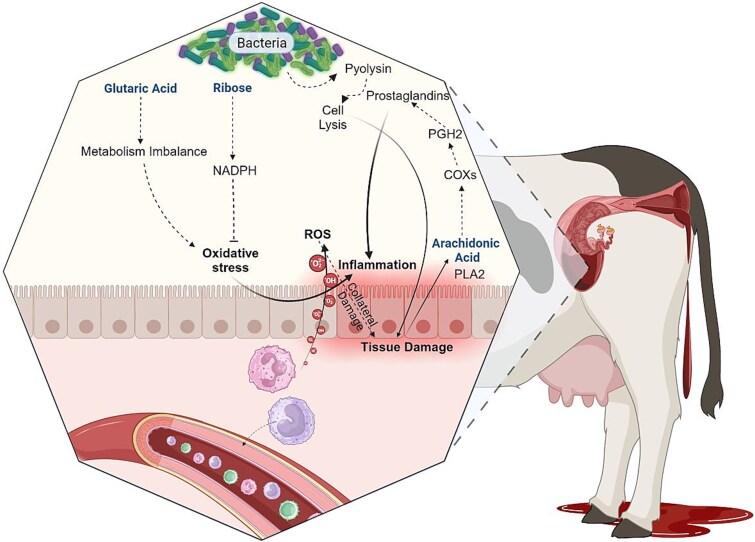

## Introduction

Metritis is a uterine disease characterized by the inflammation of all layers of the uterus due to a dysbiosis of the uterine microbiome with overgrowth of opportunistic pathogens such as *Fusobacterium*, *Bacteroides*, and *Porphyromonas*. Approximately 25% of lactating dairy cows in the U.S. are affected by metritis [1]. Multiple studies have consistently reported the detrimental impacts on cow welfare [2, 3], reproductive performance [4, 5], milk production [6, 7] and removal from the herd [8, 9] associated with metritis. Combining the economic losses linked to reduced productive and reproductive performance with previously mentioned factors and the costs associated with antimicrobial treatment, each case of metritis has been estimated to cost between US$ 156 and $949 [10–12].

Studies have reported that approximately 20% of cows treated for metritis with antimicrobials still display clinical signs of the disease two weeks after treatment, defined here as clinical cure failure of metritis [9, 13–15]. Clinical cure failure of metritis was associated with intensified negative impacts on health and performance. Cows that failed to achieve cure after treatment had increased incidence of purulent vaginal discharge and endometritis, reduced risk of resumption of estrous cyclicity, reduced risk of pregnancy by the end of 300 days postpartum (dpp), increased risk of removal from the herd, and decreased milk production within 300 dpp [9, 15]. Although the mechanisms that underlie clinical cure failure of metritis are not fully elucidated, studies reported that cows that fail to undergo clinical cure may have a more intense degree of inflammation and tissue damage compared to cows that achieve clinical cure of metritis. For instance, greater plasma concentrations of haptoglobin were associated with clinical cure failure of metritis [16, 17]. Moreover, fever defined as rectal temperature ≥39.5°C at the time of metritis diagnosis, vulvovaginal laceration, and retained fetal membranes were associated with greater odds of clinical cure failure [9, 18]. Lastly, multiple studies have depicted the differences in cow behavior, such as reduced rumination and feeding time and increased idle time associated with clinical cure failure of metritis [19, 20], illustrating the differences in condition severity between clinically cured cows and cows not cured.

Considering the additional detrimental impacts of clinical cure failure of metritis, understanding its pathophysiology would enable the development of preventative methods and potentially alleviate its subsequent detrimental impacts. Studies focused on characterizing differences in the uterine microbiome associated with clinical cure failure of metritis have reported contrasting results. For instance, Jeon et al. [21] reported differences in evenness and diversity associated with clinical cure failure of metritis 5 days after diagnosis, in addition to the greater uterine prevalence of *Fusobacterium*, *Bacteroides*, and *Porphyromonas* in cows that failed to cure regardless of antimicrobial treatment. Conversely, Figueiredo et al. [22] did not observe major differences in the uterine microbiome associated with clinical cure failure 5 days after diagnosis. To further investigate mechanisms associated with clinical cure failure, a recent study that aimed to assess differences in vaginal metabolome associated with metritis and clinical cure reported different prevalence of specific vaginal metabolites associated with amino acid metabolism and transfer RNA biosynthesis [23]. The metabolome analysis is an insightful approach to explore potential disease mechanisms, as the connection between the microbiome and the metabolome is critical, and microbial populations and host cells modulate the metabolomic composition of the uterine environment [24]. In context of the vaginal metabolome and clinical cure failure of metritis, it is possible that the differences in amino acid and tRNA biosynthesis pathways are a function of bacterial and cellular proliferation, aligning with the differences in uterine microbiome associated with clinical cure described by Jeon et al. [21].

Despite previous studies, data regarding the metabolome of the uterus and serum of cows with clinical cure failure are lacking. Considering the promising results observed by de Oliveira et al. [23], further exploration of the differences in the uterine metabolome associated with clinical cure failure of metritis may elucidate pathways and potential biomarkers. It is possible that by investigating the uterine and serum metabolomic composition from clinically and non-cured cows with metritis, we may further elucidate local and systemic mechanisms associated with uterine diseases and their progression. This study contributes to our overarching goal of early identification of cows at greater risk of clinical cure failure of metritis and the development of alternative treatment strategies. We hypothesize that the metabolome profiles of cows with clinical cure failure differ significantly in inflammation and energy metabolism pathways compared to those achieving clinical cure. The objective of this study was to characterize differences in the uterine and serum metabolome associated with clinical cure failure of metritis in lactating Holstein cows.

## Materials and methods

All procedures involving animals were approved by the animal care and use committee of the University of Florida (protocol no. 201810204).

### Study population and housing

The data used in the present study was generated by another cohort study conducted by our group in two dairy farms in Florida from February to November 2018. The number of lactating cows and herd rolling average for milk yield for each herd were 5270 and 11 000 kg (herd 1) and 2500 and 12 049 kg (herd 2). Both herds milked only Holstein cows thrice daily. The cows were fed a total mixed ration (TMR) to meet or exceed the nutritional requirements for a 650 kg cow producing 40 kg/day of 3.5% fat-corrected milk (NRC, 2001). Cows were housed in naturally ventilated freestall barns featuring sprinklers and fans positioned over the feed bunk and fans over the deep-bedded sand stalls. Additionally, the cows had ad libitum access to water. Additional information regarding the participating dairies was previously published by Figueiredo et al. [25].

### Definition of metritis, treatment, clinical cure failure, and sampling

Metritis was diagnosed based on a visual evaluation of vaginal discharge using a Metricheck device (Simcro, Hamilton, New Zealand). Cows had their vaginal discharge evaluated at 5, 7, and 11 dpp (herd 1) or 4, 6, 8, 10, and 12 dpp (herd 2). Vaginal discharge was scored using a 5-point scale: 1 = clear mucus or lochia; 2 = clear mucus with flecks of pus; 3 = mucopurulent discharge with <50% of pus; 4 = mucopurulent discharge with ≥50% of pus or without fetid reddish mucous discharge; 5 = fetid, watery, reddish-brown discharge [26]. Metritis was characterized by the presence of vaginal discharge score 5. A total of 24 cows were diagnosed with metritis (*Metritis*; *n* = 24; 17 cows in herd 1 and 7 cows in herd 2; 6 primiparous and 18 multiparous cows). Cows without metritis (vaginal discharge score ≤ 3) of similar parity and dpp were paired and served as a negative control group (*NoMetritis*; *n* = 24; 17 cows in herd 1 and 7 cows in herd 2; 4 primiparous and 20 multiparous cows). Average pairing interval between cows with metritis and their negative control counterpart (NoMetritis) was 4.2 ± 4.5 days. The day of metritis diagnosis and pairing of cows without metritis was considered study day 0. All cows diagnosed with metritis received systemic antimicrobial therapy starting on day 0. Cows were treated with either 6.6 mg/kg of body weight of ceftiofur crystalline free acid s.c. (Excede Sterile Suspension, Zoetis, Madison, NJ, USA) injected twice 72 h apart (*n* = 21; herd 1, *n* = 17; herd 2, *n* = 4) or 11 mg/kg of body weight of ampicillin trihydrate i.m. (Polyflex, Boehringer Ingelheim Vetmedica, Duluth, GA, USA) injected once daily for five consecutive days (herd 2, *n* = 3). Although different antimicrobials were used to treat cows with metritis in this study, the formulations used herein produced similar clinical and productive outcomes [27, 28] and similar effects on the uterine microbiome [21] in cows with metritis based on previous studies. Incidences of metritis in the two dairy farms used in this study were 20% for herd 1 and 19% for herd 2. Clinical cure failure following antimicrobial therapy was defined for cows with metritis still displaying vaginal discharge score 5 on day 5 (*NoCure*; *n* = 16: 12 cows in herd 1 and 4 cows in herd 2; 4 primiparous and 12 multiparous cows), whereas clinical cure was defined for cows with vaginal discharge scores ≤4 on day 5 (*Cure*; *n* = 8: 5 cows in herd 1 and 3 cows in herd 2; 2 primiparous and 6 multiparous cows). Day 5 was selected for the evaluation of clinical cure of metritis and subsequent sample collection because a recent study has reported differences in the uterine microbiome associated with clinical cure failure of metritis in the same period [21], and the uterine microbiome between cows with and without metritis is similar 9 days after diagnosis and treatment [29].

Before administering antimicrobials on day 0, a single technician performed a low-volume uterine lavage in all cows. Briefly, the vulva was cleaned with a paper towel and ethanol (70% vol./vol.), and a single-use plastic round-tip pipette (UterFlush pipettes, Van Beek, Orange City, IA, USA) was introduced into the vagina and manipulated through the cervix. A total of 30 mL of sterile saline solution (0.9% sodium chloride irrigation, Baxter, Deerfield, IL, USA) was infused into the lumen of the uterine body using a 60 mL syringe (Covidien, Mansfield, MA, USA). At least 30 mL of uterine contents were homogenized, retrieved into the same 60 mL syringe, and transferred to a sterile 15 mL conical tube (VWR, Radnor, PA, USA). Blood samples were collected on day 0 by puncture of the coccygeal vein using a vacutainer tube without anticoagulant (Vacutainer, Becton Dickinson). A second uterine lavage was performed on day 5 in all cows following the same procedures described. Tubes were placed on ice immediately after collection and transported to the laboratory within 6 h of collection. Uterine samples were homogenized and aliquoted into multiple 2 mL microcentrifuge tubes (Eppendorf, Enfield, CT, USA) and stored at −80°C until assayed. Blood tubes were centrifuged at 4000 × g for 15 min at 4°C, and serum was harvested, aliquoted into 2 mL microcentrifuge tubes, and stored at −80°C until assayed.

### Metabolome analyses

Uterine lavage and serum samples were sent to the West Coast Metabolomics Center at the University of California-Davis for metabolome analyses. Briefly, Matyash extraction procedure, including MTBE, MeOH, and H_2_O was performed to remove large lipid molecules from samples. Samples were shaken at 30°C for 1.5 h, followed by the injection of 91 μL of MSTFA + FAMEs, and shaken at 37°C for 30 min to finish derivatization. The samples were analyzed in a single batch using untargeted gas chromatography with time-of-flight mass spectrometry (GC-TOF-MS). A column containing 95% dimethyl / 5% diphenyl polysiloxane was used, with helium as the carrier gas. The column temperatures ranged from 50°C to 330°C at a 1 mL/min flow rate. Initially set at 50°C, the oven temperature was increased by 20°C per minute until it reached 330°C, which was maintained for 5 min. The injection temperature started at 50°C and increased to 250°C in 12°C per second increments. Primary metabolite (amino acids, hydroxyl acids, carbohydrates, sugar acids, sterols, aromatics, nucleosides, amines, and various compounds) retention was assessed based on their mass and charge relationship, using the default settings from ChromaTOF v. 2.32. Peak heights were determined for each metabolite. Metabolites were annotated using the PubChem (https://pubchem.ncbi.nlm.nih.gov/), Kyoto Encyclopedia of Genes and Genomes (https://www.genome.jp/kegg), and Human Metabolome (https://hmdb.ca/) databases.

### Sample size and statistical analyses

Sample size calculation was not performed as the data used herein was generated by another study that aimed to assess the differences in the uterine and serum metabolome associated with metritis [25].

Three separate datasets (uterus day 0, serum day 0, and uterus day 5) containing only detectable metabolites and peak heights were obtained via GC-TOF-MS. A total of 1174 unique metabolites were detected across the three datasets; however, only 217 were annotated based on the aforementioned databases (Supplemental Table S1). Only annotated metabolites (*n* = 217) were used for subsequent statistical analyses. Metabolomic analyses were conducted using MetaboAnalyst 6.0 [30]. Before analyses, residue distribution was visually evaluated, and data were auto-scaled and transformed if necessary. No missing values were detected prior to analysis. Differences in the uterine (day 0 and day 5) and serum (day 0) metabolome between the groups (Cure, NoCure, and NoMetritis) were assessed using partial least squares–discriminant analysis (PLS-DA) coupled with permutational MANOVA using 2000 permutations. The PLS-DA test was chosen due to its capacity to assess the interrelationship between metabolites and the study groups. Prior to MANOVA, cross-validation (CV) was conducted using a 5-fold CV method for five maximum components and performance was measured by the Q2 values. ANOVA coupled with Fishers LSD and hierarchical clustering heat maps and dendrograms (Euclidean distance and Ward clustering method) were also performed when permutational MANOVA was statistically significant (*P* ≤ 0.05). Cows without metritis (NoMetritis) were included in all analyses for completeness and separation of effects of metritis in models.

If differences in either serum or uterine metabolome associated with clinical cure failure were observed, enriched pathway analysis was performed to identify affected metabolic pathways based on the Kyoto Encyclopedia of Genes and Genomes database for Bos taurus using MetaboAnalyst 6.0. All *P*-values obtained from ANOVA and enriched pathway analyses were adjusted for false discovery rate (FDR; [31]) to account for multiple comparisons. Differences with adj. *P* ≤ 0.05 were considered statistically significant for all analyses except for enriched pathway analysis, which was considered adj. *P* ≤ 0.10.

**Figure 1:**
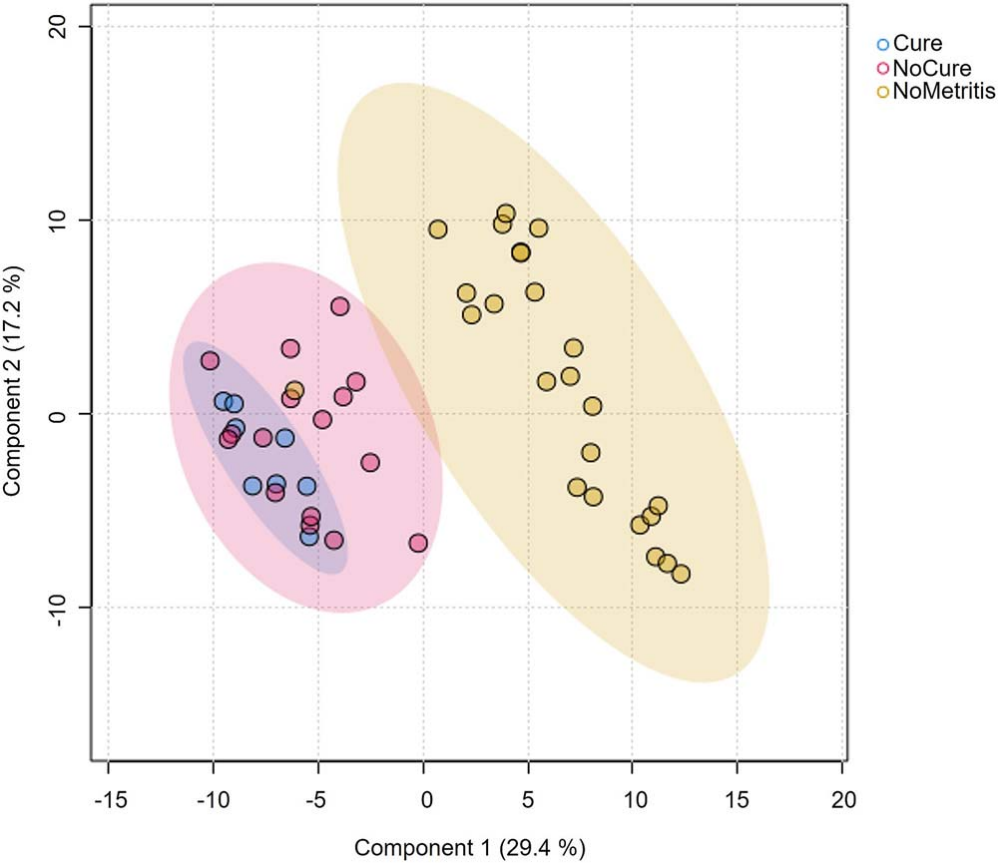
Comparison of uterine metabolome on day 0 between cured cows (Cure; *n* = 8), non-cured cows (NoCure; *n* = 16), and cows not diagnosed with metritis (NoMetritis; *n* = 24). The day of metritis diagnosis and pairing was considered study day 0. Partial least square-discriminant analysis (PLS-DA); *P* < 0.01 for permutational multivariate ANOVA with 2000 permutations.

## Results

### Differences in uterine metabolome associated with clinical cure failure of metritis

Average enrollment dpp and lactation for cows in Cure, NoCure, and NoMetritis are 7.7 and 3.4, 5.5 and 3.0, and 6.4 and 2.8, respectively. A total of 187 annotated metabolites were identified by GC-TOF-MS in uterine samples collected on day 0 and day 5 (Supplemental Table S1).

**Figure 2:**
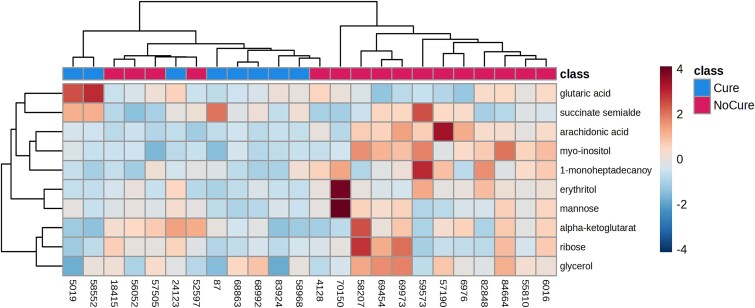
Hierarchical clustering heat maps depicting the 10 differently prevalent metabolites associated with clinical cure failure of metritis on day 0. Each colored cell on the map corresponds to fold differences, with metabolites in rows and cows/sample in columns

**Figure 3:**
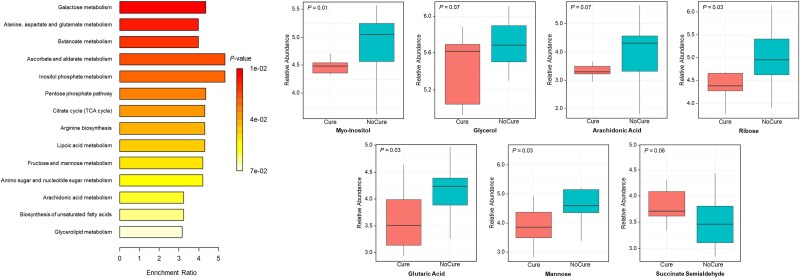
Metabolic pathways associated with clinical cure failure of metritis on day 0 based on enriched pathway analysis and relative abundance of metabolites associated with clinical cure failure of metritis that compose metabolic pathways. *P*-values adjusted for multiple comparisons using false discovery rate (FDR) on enriched pathway analysis

On day 0, differences (*P* < 0.01) in the uterine metabolome were observed between Cure, NoCure, and NoMetritis based on permutational MANOVA and PLS-DA (Figure 1, Supplemental Figure 1). A total of 110 metabolites (Adj. *P* < 0.05; Supplemental Table S2) were identified by ANOVA on day 0. However, only 10 metabolites were differently abundant in the uterus of Cure compared with NoCure cows (Table 1, Figure 2). Enriched pathway analysis using 10 metabolites associated with clinical cure failure of metritis were conducted. Only seven metabolites were connected to metabolic pathways, which were linked primarily to carbohydrate, amino acid, and lipid metabolism (Figure 3; Supplemental Table S3).

**Table 1:** Uterine metabolites associated with clinical cure failure of metritis on the day of diagnosis (day 0).

*Metabolite*	**Adj. *P*** ^**1**^	**Fishers LSD** ^**2**^	
		*Metritis vs. NoMetritis*	*Cure vs. NoCure*
Myo-Inositol	<0.01	1	1
Glycerol	<0.01	1	1
Arachidonic Acid	<0.01	1	1
Erythritol	<0.01	1	1
Ribose	<0.01	1	1
Alpha-Ketoglutarate	<0.01	1	1
1-Monoheptadecanoyl Glyceride	<0.01	1	1
Succinate Semialdehyde	<0.05	1	1
Glutaric Acid	<0.05	1	1
Mannose	<0.05	1	1

^1^ *P*-values from ANOVA coupled with Fishers LSD were adjusted for false discovery rate (FDR).

^2^Dichotomous description of differences according to metritis and cure, where 1 indicates differences associated with metritis or cure, and 0 indicates lack of differences associated with metritis or cure.

On day 5, permutational MANOVA and PLS-DA were not statistically significant (*P* = 0.88), indicating that the uterine metabolome between Cure, NoCure, and NoMetritis was similar. ANOVA was not performed.

### Serum metabolome associated with clinical cure failure of metritis

**Figure 4:**
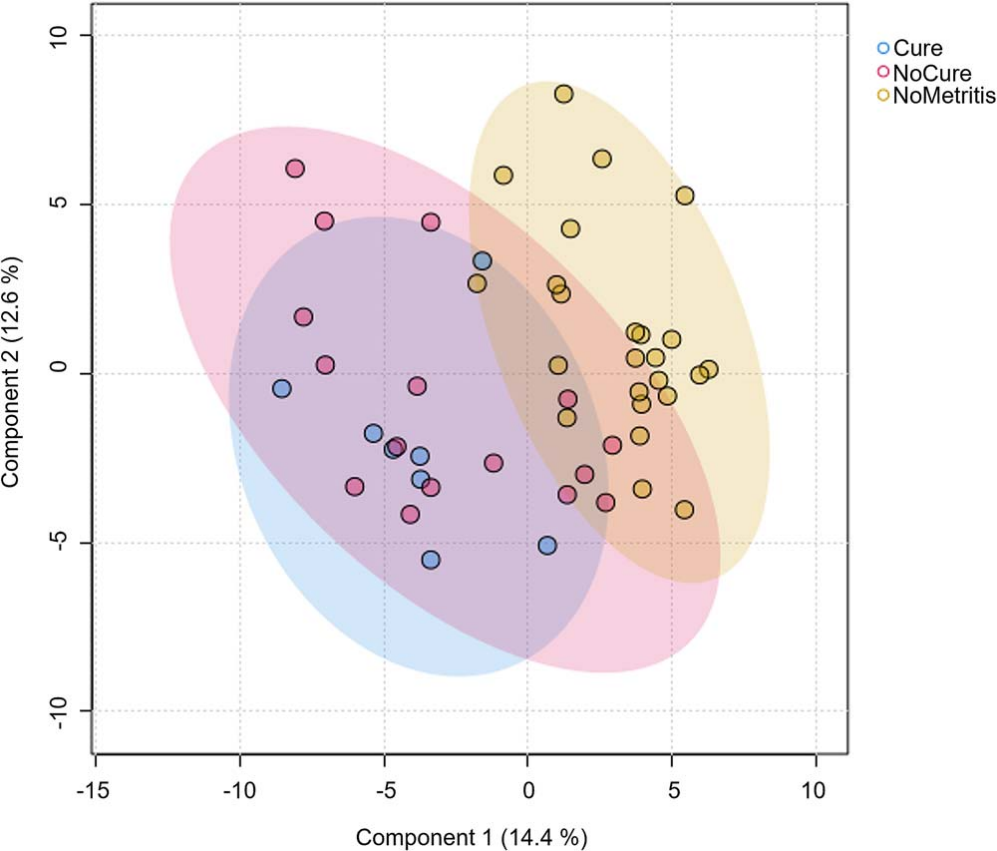
Comparison of serum metabolome on day 0 between cured cows (Cure; *n* = 8), non-cured cows (NoCure; *n* = 16), and cows not diagnosed with metritis (NoMetritis; *n* = 24) paired based on parity and calving date. The day of metritis diagnosis and pairing was considered study day 0. Partial least square-discriminant analysis (PLS-DA); *P* = 0.03 for permutational multivariate ANOVA with 2000 permutations.

A total of 152 annotated metabolites were identified by GC-TOF-MS in serum samples collected on day 0 (Supplemental Table S1). Differences (*P* = 0.03) in the serum metabolome on day 0 between Cure, NoCure, and NoMetritis were observed based on permutational MANOVA and PLS-DA (Figure 4). A total of 10 metabolites (Adj. *P* < 0.01; Table 2) were identified by ANOVA on day 0. Results from Fishers LSD indicated that the differences in the serum metabolome observed on day 0 were associated with metritis rather than the clinical cure failure of metritis.

**Table 2:** Serum metabolites associated with metritis on the day of diagnosis (day 0).

*Metabolite*	**Adj. *P*** ^**1**^	**Fishers LSD** ^**2**^	
		*Metritis vs. NoMetritis*	*Cure vs. NoCure*
Glutaric Acid	<0.01	1	0
5-Aminovaleric Acid	<0.01	1	0
Citric Acid	<0.01	1	0
Isocitric Acid	<0.01	1	0
Citramalic Acid	<0.01	1	0
Succinic Acid	<0.01	1	0
Piperidone	<0.01	1	0
2-Hydroxybutanoic Acid	<0.01	1	0
Mannose	<0.01	1	0
2-Hydroxyglutaric Acid	<0.01	1	0

^1^ *P*-values from ANOVA coupled with Fishers LSD were adjusted for false discovery rate (FDR).

^2^Dichotomous description of differences according to metritis and cure, where 1 indicates differences associated with metritis or cure, and 0 indicates lack of differences associated with metritis or cure.

## Discussion

This study aimed to characterize differences in uterine and serum metabolome associated with clinical cure failure of metritis in lactating Holstein cows. Because of the severe differences in cow performance and health associated with clinical cure failure of metritis, and previously published data by de Oliveira et al. [23], we hypothesized that the uterine and serum metabolome of cows with clinical cure failure of metritis differs from that observed in cows that achieved clinical cure. On the day of metritis diagnosis (day 0), the uterine metabolome differed between cows with and without metritis, which is unsurprising and has been described by other authors [23, 25].

Within the group of cows with metritis, those that underwent clinical cure had specific differences in the uterine metabolome on day 0 compared with cows that did not cure. The serum metabolome differed slightly between cows with and without metritis on day 0, which has been previously described and discussed [32–34]. However, no differences were associated with clinical cure failure. Surprisingly, no differences in the uterine metabolome on day 5 were associated with clinical cure failure of metritis in dairy cows.

On the day of metritis diagnosis (day 0), 10 uterine metabolites were associated with clinical cure failure of metritis (assessed 5 days after diagnosis; day 5). Among the differently prevalent metabolites associated with clinical cure failure are arachidonic acid, ribose, and glutaric acid, which contributed to the upregulation of the arachidonic acid metabolism, biosynthesis of unsaturated fatty acids, pentose phosphate metabolism, and citrate cycle (TCA) pathways. In this study, cows with clinical cure failure had a greater concentration of arachidonic acid in the uterus on day 0 than cured cows. Arachidonic acid, a key component of phospholipids that compose the eukaryotic cell membranes, is released subsequent to enzymatic or traumatic action toward such cells [35]. Upon release from phospholipids, arachidonic acid in freeform is metabolized by cyclooxygenases to promote the secretion of several inflammatory cytokines and thromboxane, or lipoxygenases for the secretion of leukotriene, or cytochrome P450 for different acids [36]. In human medicine, arachidonic acid has been used as a biomarker for inflammation [37]. In the context of the cow, it could indicate that cows that fail to undergo clinical cure are under a greater uterine inflammatory process and tissue damage. The mechanisms associated with such inflammatory processes and tissue damage have not been completely elucidated, and it is possible that they are mediated by bacterial or immune cell functions. For instance, pyolysin, which is an enzyme produced by a prevalent bacterium in the uterus of cows with metritis (*Trueperella pyogenes;* [38]), has been associated with increased endometrial cell damage [39]. Studies evaluating the uterine microbiome associated with clinical cure failure of metritis are scarce and contradictory [21, 22]. For instance, greater uterine prevalence of *Fusobacterium*, *Bacteroides*, and *Porphyromonas* was associated with clinical cure failure of metritis [21], but no major differences between groups were observed by another study [22]. Nevertheless, more uterine microbiome studies are warranted to deeply explore shifts in microbial communities associated with metritis and bacterial function, considering that previous datasets were 16S rRNA sequencing-based. Although biological processes associated with the uterine immune response towards bacterial infections have not been deeply explored, one study reported a great influx of polymorphonuclear cells (PMN) upon T. pyogenes infusion in the uterine lumen of dairy cows [40]. Part of the mechanisms of PMN activation involves the production of reactive oxygen species, which may be beneficial for bacterial clearing; however, it may cause detrimental impacts on host cells upon inadequate regulation of its pathways, generating an excessive inflammatory process [24, 41, 42]. In addition, a study reported that cows with metritis had higher plasma malondialdehyde concentrations, a marker of oxidative stress, compared to healthy cows [43]. This suggests that cows with clinical cure failure may potentially experience greater uterine oxidative stress and inflammatory processes. Similarly to arachidonic acid, glutaric acid, and ribose have also been associated with upregulated inflammatory processes [44] and energy production [45]. The specific differences in uterine metabolic profiling associated with clinical cure failure may illustrate a greater degree of inflammation, which corroborates with previously published data. For instance, cows that failed to undergo a clinical cure of metritis had greater blood haptoglobin levels [16, 17], fever [9, 18], and vaginal laceration scores [18].

Contrary to our hypothesis, we did not observe any differences in the serum metabolome on day 0 associated with clinical cure failure. Serum metabolomic differences observed in the current study were associated with metritis only, which could possibly indicate that clinical cure is more tightly related to biological processes in the uterus rather than systemic processes. Nevertheless, previous studies that reported differences in blood metabolite concentration associated with clinical cure failure used a different approach for analysis compared to our study. For instance, Machado et al. [16] and Menta et al. [17] reported differences in serum and plasma haptoglobin, creatinine, triglycerides, cholesterol, and total protein associated with clinical cure failure using targeted methods for metabolite quantification. Herein, we implemented an untargeted method to identify and quantify metabolites, which could play a role in the differences in outcomes observed. Other studies that evaluate the serum metabolome of cows with and without metritis also reported contrasting outcomes according to the metabolomic method implemented [25, 32, 33]. Nevertheless, no differences in serum metabolome were observed between cows with and without metritis [25] using the same metabolomic approach used herein. This could indicate that although there are specific differences in serum metabolome associated with metritis and clinical cure failure (captured by targeted metabolomic techniques), such differences do not translate into significant differences in the overall serum composition.

Surprisingly, differences associated with clinical cure failure on day 0 did not persist until day 5 (the day of cure assessment). The similarity in uterine metabolome between clinically cured and non-cured cows opposes the findings reported by de Oliveira et al. [23]. Although the method of metabolite identification and quantification (including the laboratory) used by de Oliveira et al. [23] is the same as used in the present study, critical differences related to study design could explain the contrast between studies. For instance, vaginal samples from a total of 17 cows with metritis in California and Texas (10 ceftiofur-treated and 7 non-treated cows), of which 5 did not undergo clinical cure by day 5, were used in the previous study [23]. Whereas 24 cows with metritis in Florida (all treated with antimicrobials), of which 16 did not undergo clinical cure by day 5 were used in this study. In addition to the sample source and sample size differences between the studies, the effects of antimicrobial treatment on the vaginal metabolome were not explored by de Oliveira et al. [23]. Although not directly related to clinical cure, three studies reported differences in the uterine microbiome of cows with metritis associated with antimicrobial treatment [21, 46, 47]. Considering that bacteria produce a portion of metabolites in the reproductive tract of cows and considering the effects of antimicrobials on bacterial communities of the uterus, it is possible that the disparities in treatment protocol between de Oliveira et al. [23] and ours explain the differences in outcomes observed. Nonetheless, the uterine microbiome associated with clinical cure failure of metritis was previously investigated by Figueiredo et al. [22] using the same samples (cows and time of collection) used herein, and no major differences in the uterine microbiome were associated with clinical cure failure on day 0 or 5. A recent study depicted the uterine progression of cows with metritis treated with non-steroidal anti-inflammatory (NSAID). A similar proportion of cows with metritis treated with NSAID underwent clinical cure 3 days after diagnosis (determined as lack of fever or depressed attitude) compared with cows treated with ceftiofur [48]. This suggests that clinical progression of metritis is a complex event that may be somewhat independent of the uterine microbiome, possibly more tightly related to the immunological response towards pathobionts, as proposed by Sheldon et al. [49], and perhaps such differences are subtle enough to be captured by untargeted metabolomic methods.

It is essential to highlight that we did not perform sample size calculations, which can restrict our capacity to identify minor differences among groups. In addition, cows in the study were located in two dairy herds in the southeast region of the U.S. (particular environmental conditions; herd and parity variability were not accounted in models), sampling collection was conducted differently than previous metabolomic studies, and all cows with metritis received antimicrobial treatment. This study highlights the need to further optimize identification of metabolites in datasets generated by untargeted methods, as a limited number of metabolites were included in this dataset (only annotated metabolites) and therefore could have limited our capacity to further understand biological processes associated with uterine diseases. Further studies are warranted to assess the effects of the interaction between metritis, clinical cure failure, and antimicrobial treatment on the uterine metabolome, using a larger and more regionally diverse dataset. In addition, more studies on the immunological status and function associated with clinical cure failure would be beneficial for a better understanding of its underlying mechanisms and possibly developing preventative measures and practical diagnostic tools based on biomarker identification and quantification.

## Conclusion

The distinct uterine metabolic profile associated with clinical cure failure of metritis observed on the day of metritis diagnosis provided valuable insights into the pathways associated with clinical cure failure. No differences associated with clinical cure failure were observed in serum metabolome on the day of metritis diagnosis. Data presented herein indicates that clinical cure failure is associated with a greater uterine inflammatory process that did not persist until cure assessment day (5 days after diagnosis). These findings highlight the complex interplay between inflammatory and metabolic pathways and possible immunological responses, underscoring the need for further research to fully elucidate this relationship. Overall, metabolomic studies could be used for biomarker identification to possibly develop early diagnostic tools for clinical cure failure of metritis in dairy cows.

## Supplementary Material

BOR_2024_0589_Pereira_Supplemental_Figure_S1_ioaf038

BOR_2024_XXXX_Pereira_Supplemental_Table_S1_ioaf038

BOR_2024_XXXX_Pereira_Supplemental_Table_S2_ioaf038

BOR_2024_XXXX_Pereira_Supplemental_Table_S3_ioaf038

## Data Availability

The data is available in the article, online supplementary material, and in open access public database Figshare (https://figshare.com/articles/dataset/BIOLRE_2024-0589_Pereira_Dataset_xlsx/28127762?file=51456329).
