# Supplementary figures and images for: Differences in uterine and serum metabolome associated with clinical cure failure of metritis in dairy cows
Source: Biol Reprod. 2025 Feb 23;112(5):858–66. doi: 10.1093/biolre/ioaf038 (PMC12078077; doi:10.1093/biolre/ioaf038)

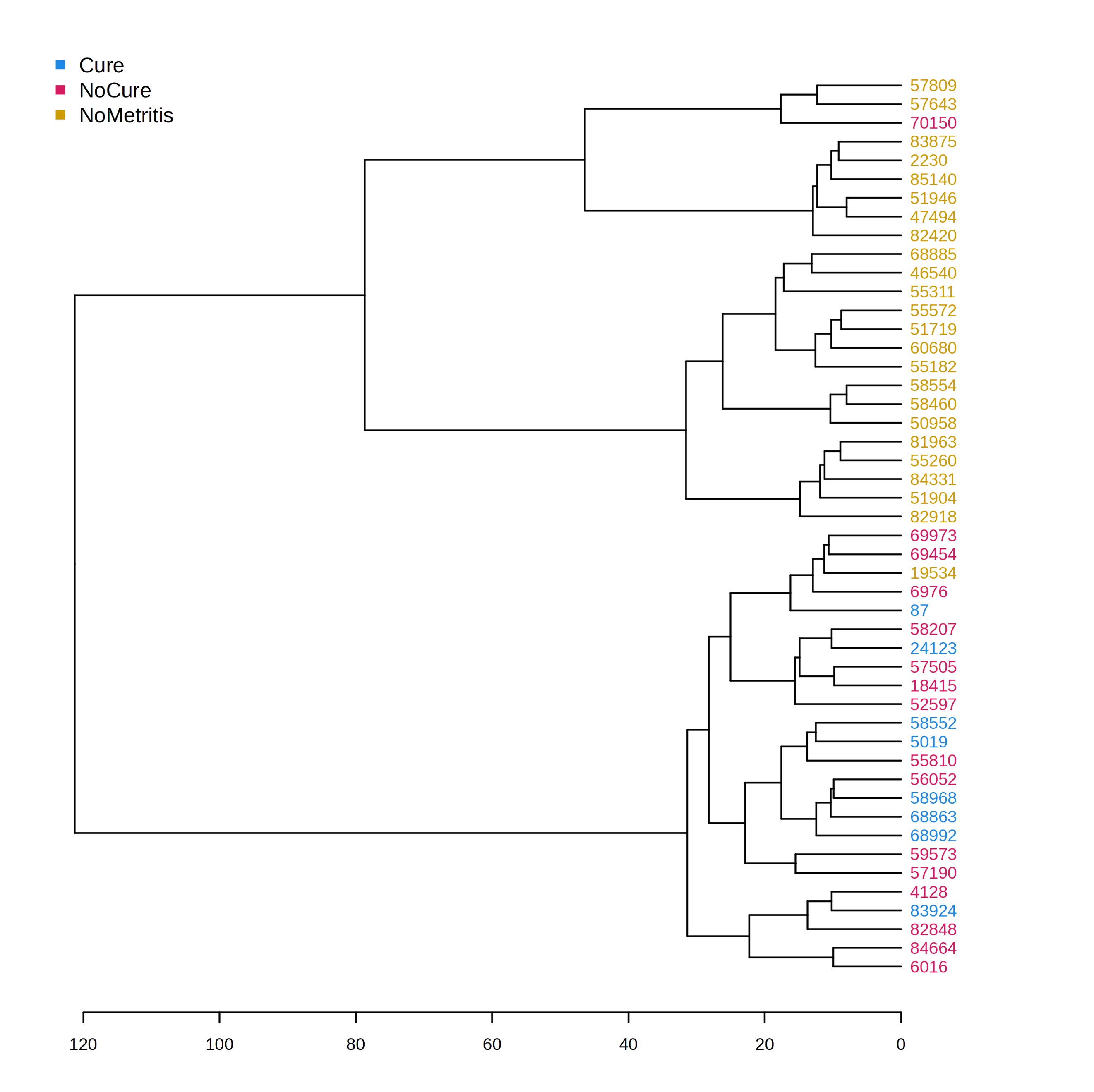

Supplement: BOR_2024_0589_Pereira_Supplemental_Figure_S1_ioaf038 [file bor_2024_0589_pereira_supplemental_figure_s1_ioaf038.jpeg]
